# Smartphone-Based Ecological Momentary Assessment of Well-Being: A Systematic Review and Recommendations for Future Studies

**DOI:** 10.1007/s10902-020-00324-7

**Published:** 2020-10-23

**Authors:** Lianne P. de Vries, Bart M. L. Baselmans, Meike Bartels

**Affiliations:** 1 Department of Biological Psychology, Vrije Universiteit Amsterdam, Van der Boechorststraat 7, 1081 BT Amsterdam, The Netherlands; 2 Amsterdam Public Health Research Institute, Amsterdam University Medical Centres, Amsterdam, The Netherlands; 3 Institute for Molecular Bioscience, The University of Queensland, Brisbane, QLD Australia

**Keywords:** Well-being, Happiness, Ecological momentary assessment, EMA, Smartphone, Passive sensing

## Abstract

Feelings of well-being and happiness fluctuate over time and contexts. Ecological Momentary Assessment (EMA) studies can capture fluctuations in momentary behavior, and experiences by assessing these multiple times per day. Traditionally, EMA was performed using pen and paper. Recently, due to technological advances EMA studies can be conducted more easily with smartphones, a device ubiquitous in our society. The goal of this review was to evaluate the literature on smartphone-based EMA in well-being research in healthy subjects. The systematic review was conducted according to the Preferred Reporting Items for Systematic Review and Meta-Analysis (PRISMA) guidelines. Searching PubMed and Web of Science, we identified 53 studies using smartphone-based EMA of well-being. Studies were heterogeneous in designs, context, and measures. The average study duration was 12.8 days, with well-being assessed 2–12 times per day. Half of the studies included objective data (e.g. location). Only 47.2% reported compliance, indicating a mean of 71.6%. Well-being fluctuated daily and weekly, with higher well-being in evenings and weekends. These fluctuations disappeared when location and activity were accounted for. On average, being in nature and physical activity relates to higher well-being. Working relates to lower well-being, but workplace and company do influence well-being. The important advantages of using smartphones instead of other devices to collect EMAs are the easier data collection and flexible designs. Smartphone-based EMA reach far larger maximum sample sizes and more easily add objective data to their designs than palm-top/PDA studies. Smartphone-based EMA research is feasible to gain insight in well-being fluctuations and its determinants and offers the opportunity for parallel objective data collection. Most studies currently focus on group comparisons, while studies on individual differences in well-being patterns and fluctuations are lacking. We provide recommendations for future smartphone-based EMA research regarding measures, objective data and analyses.

**Electronic supplementary material:**

The online version of this article (10.1007/s10902-020-00324-7) contains supplementary material, which is available to authorized users.

Feelings of well-being and happiness play a preventive role in psychopathology (e.g. depression) and are important to overall physical and mental health (Diener et al. 2017; Greenspoon and Saklofske 2001; Howell et al. 2007). Happier and more optimistic people are found to live longer and healthier lives (Kim et al. 2019; Steptoe 2019; Zaninotto and Steptoe 2019). For instance, feeling happy reduces the likelihood of a heart disease and leads to better cardiovascular health (Boehm et al. 2012; Davidson et al. 2010). Furthermore, well-being is associated with successful outcomes in life, e.g. happier people are more often married, have better relationships with their partners and are more socially engaged (Lyubomirsky et al. 2005; Moore and Diener 2019). The effects of well‐being on health are found to be independently of the negative effects of ill‐being on health, indicating the importance of investigating well-being (Howell et al. 2007).

Well-being is defined in multiple ways in the literature and often a distinction between subjective and psychological well-being is made (Keyes et al. 2002). Briefly, subjective well-being is characterized by high levels of positive affect, low levels of negative affect and a higher subjective evaluation of life satisfaction (Diener et al. 2018), whereas psychological well-being refers to thriving, positive functioning, and judgments about the meaning and purpose of an individual’s life (Ryff 1989). As subjective and psychological well-being are strongly related (e.g. Baselmans et al. 2019; Joshanloo 2016), in this paper, we refer to well-being in the broad sense, including all definitions and constructs.

The majority of studies assessing well-being (WB) make use of questionnaires that are completed by participants at a single time point or multiple times (see for a review of well-being questionnaires and well-being research Cooke et al. 2016; Diener et al. 2012; Linton et al. 2016). Well-being questionnaires ask about the general well-being or happiness and include for example the Life Satisfaction Scale (Diener et al. 1985), the Cantril ladder (Cantril 1965), or the Subjective Happiness Scale (Lyubomirsky and Lepper 1999). The scores on these well-being measures are found to be relatively stable and reliable over time (e.g. Fujita and Diener 2005; Pavot 2008; Schimmack and Oishi 2005).

However, like many complex human traits, momentary feelings of well-being (e.g. mood) fluctuate over time and in different contexts (Eid and Diener 2004; Li et al. 2014; Lyubomirsky 2001). Individuals can have similar well-being scores at different questionnaire waves, while the underlying pattern (WB over the day or week) differs substantially. Some people show relatively stable levels of WB over the day or week, while others fluctuate a lot (Eid and Diener 1999; Gadermann and Zumbo 2007). To better understand the relationship between well-being and for instance psychopathology or environmental influences, it is important to understand within person fluctuations of well-being over time. One way to capture the dynamic nature of well-being is by measuring well-being multiple times a day in the natural context and daily life of participants, i.e. using an ecological momentary assessment design.

Investigating daily experiences and behavior of people in their natural context is not a novel idea. Already in the 1920’s, the dairy method was used by Favill and Rennick (1924) and Flügel (1925) to collect reports of participants about their symptoms, behavior or mood over several days. In different disciplines (e.g. behavioral medicine and psychology), these methods developed further and are nowadays known under different labels, such as ambulatory assessment (Fahrenberg 1996; Fahrenberg et al. 2007), the experience sampling method (ESM: Csikszentmihalyi and Larson 1987) and ecological momentary assessment (EMA: Stone and Shiffman 1994). The goal of all these assessment methods is to study people in their natural environment, including measures of self-report, observational, biological, physiological and behavioral measures. Later, Kahneman and colleagues (2004) developed the day reconstruction method (DRM), where participants are asked to reconstruct and describe all experiences and events of the day. See Wilhelm et al. (2012) for a detailed review and historical overview of the development of the different methods. In this review we will use the term ecological momentary assessment (EMA) to describe all ambulatory assessment, ESM and EMA methods, as this term is widely used in well-being research.

Before technological developments, EMA studies required participants to carry beepers and booklets of questionnaires. After each random timed beep, participants had to complete the questions using pen and paper. In an early study and small sample, Dysinger (1938) reported individual differences in the average level of mood and the scale of fluctuations. However, averaged across participants, there was no evidence for daily or weekly periodicity in these fluctuations. Later, after technological developments, devices such as personal data assistants (PDA) and palmtop computers were introduced to collect EMA data. Data collection became easier and according to the results of larger EMA studies, on average, happiness varied both throughout the day (e.g. happier in the afternoon compared to the morning) and the week (e.g. happier on a Saturday compared to Monday) (e.g. Brandstatter 1991; Csikszentmihalyi and Hunter 2003; Zelenski and Larsen 2000). Furthermore, physical, social and leisure activities were associated with higher degrees of happiness, whereas being alone or at work was associated with lower happiness (Csikszentmihalyi and Hunter 2003; Schwerdtfeger et al. 2008) and smoking was unrelated to momentary positive affect (Shiffman et al. 2002).

Levering the rapid technological progression in the last few years, EMA researchers have started to replace the older methods by smartphone applications (Runyan and Steinke 2015). Using smartphones can lead to a leap forward in EMA research, since smartphones are ubiquitous in our society and data can be collected more easily. However, smartphone-based EMA research does also lead to problems and difficulties. In this paper, we first describe smartphone-based EMA studies and their advantages and difficulties. Furthermore, we systematically review the literature on smartphone-based ecological momentary assessment of well-being in healthy participants.

Since the different well-being measures show a strong phenotypic and genetic overlap and the field of smartphone-based EMA research is relatively new, we will include all measures of well-being (e.g. happiness, positive affect, life satisfaction, quality of life). Specific research questions addressed in our review are: What are the used designs (context, schedule, sampling, WB measure, applications, smartphones and statistical analyses) in smartphone-based EMA studies to well-being? To what extent is objective data, such as GPS or accelerometer data included in smartphone-based EMA well-being research? Is the response rate and compliance in smartphone-based EMA studies related to the design? What are the results of smartphone-based EMA studies with respect to well-being? Finally, what are the limitations in current smartphone-based EMA? Based on guidelines proposed by Liao et al. (2016), we will describe the studies in the areas of (1) sampling and measures, (2) schedule, (3) objective data, (4) technology and administration, (5) prompting strategy, and (6) response and compliance. Additionally, we will (7) describe the analyses, (8) summarize the findings and (9) report limitations and risk of bias in the reviewed studies. In addition, as smartphone-based EMA designs are comparable to studies using other devices to collect EMA data, such as palmtop computers or PDAs, we compare the designs and findings of smartphone-based and other device-based EMA studies. Lastly, we provide guidelines for using (smartphone-based) EMA in future well-being research based on the findings.

## Smartphone-Based EMA Studies

Recently, more researchers are applying EMA designs in their studies, since the development of smartphones and applications facilitate the use of such designs. Nowadays, smartphone research is feasible for widespread use, especially in the more economically developed countries. The number of mobile phone users worldwide is reaching 5.2 billion, with 3.5 billion people owning a smartphone, i.e. more than one third of the population (GSMA intelligence, 2019). In the Western world and USA, already more than 70% of the population owns a smartphone. Furthermore, the development of tailored EMA applications becomes easier nowadays, as more knowledge and app building software becomes available.

The characterizing feature of EMA studies is the design in which daily behavior and experiences are assessed multiple times per day. EMA data have a high ecological validity, since information is collected in the moment of the experience and memory or recall biases are reduced (Schwarz 2007; Scollon et al. 2003). Different forms of sampling can be used (Shiffman et al. 2008). In time-contingent sampling designs, participants are signaled to answer questions at fixed times or random times within a predefined time frame. In interval-contingent sampling designs participants are prompted to fill in questions after a time interval has passed. In event-contingent sampling designs, participants are requested to complete questions when a predefined event happens. The latter can be subjective and initiated from the participant him- or herself (e.g. when drinking alcohol) or objective (e.g. at a specific GPS location or a specific level of physical activity). Mixed designs combine event and time or interval sampling (e.g. to prevent too little data points).

### Advantages and Difficulties

Integrating smartphone applications into (EMA) research has multiple advantages compared to pen-and-paper methods and older EMA devices, such as PDAs (García et al. 2016).Whereas pen-and-paper methods could not measure the compliance with the scheduled assessment times, response logging is automated in applications, resulting in specific response time information. Second, large nation- or worldwide samples, even in more remote areas, can be reached with EMA applications, as researchers do not need to provide participants with PDAs or palm-top computers anymore. The application can also be used longitudinally. Third, EMA applications can be more easily designed and optimized according to the research question. Related, several sensors of smartphones can be used to measure passive data continuously, for example location, light and noise levels, accelerometer and gyroscope data and phone use. Adding the data of these mobile sensors to a smartphone-based EMA study is relatively easy and valuable. For example, instead of asking participants to report their physical activity or phone use, we can infer this from the continuous stream of accelerometer and screen use data, resulting in objective and rich data.

The nature of EMA data (i.e. real-time and real-life data) allows to accurately capture the variability of subjective experiences and to detect and discover patterns, which are missed when using a sum or average score. For example, in the field of physical activity, an accelerometer study of Chinapaw et al. (2019) suggest that not the volume, but the individual pattern of accumulated physical activity is important in relation to health. Similar for EMA data, using advanced analyses (e.g. time-series analyses) can lead to new insights in patterns and fluctuations. EMA data are powerful to make inferences for an individual instead of for the (often non-existent) average person. Since subjective experiences are measured multiple times a day, the intra-individual variability can be assessed and related to other variables. For example, in a small sample of depressed and non-depressed participants, Stavrakakis et al. (2015) measured physical activity and affect multiple times per day and investigated the fluctuations for each participant. Individual differences in both the daily pattern and the strength and direction of the relationship between physical activity and affect were found, indicating important inter-individual variability. Furthermore, Wichers et al. (2016) provided evidence for an complex dynamic pattern and network of mental states underlying depression using extensive EMA data in one participant.

Designing smartphone-based EMA studies can also lead to difficulties. The main problems are issues with (personal) privacy, the intrusive design and data storage (Trull and Ebner-Priemer 2013). Especially when combining EMA with passive sensor data, privacy and data storage are important as large amounts of personal data are collected. Passive data contains highly sensitive information and possibly identifying personal data, such as GPS location. The question is whether such data can be fully anonymized, as combining data of multiple passive measures might lead to identifying information about participants. For example, Brownstein et al. (2006) could identify the home locations for 79% of the participants in a study, based on a published image map. This clearly violates the privacy of participants. Researchers should put effort in anonymizing data and use for example the relative location data to protect privacy. Next, multiple assessments can result in a high daily burden for participants in smartphone-based EMA studies, leading to increased drop outs and decreased response rates (Shiffman et al. 2008). Related, most studies do not pay participants for their participation. The trade-off between participating and the individual benefit might be unclear for participants, leading to less willingness to participate. Lastly, the multiple assessments and passive data collection can have an effect on the battery life of (older) smartphones, affecting daily life or excluding part of the population.

## Methods

### Systematic Review of Smartphone-Based EMA Studies

#### Eligibility Criteria

To review the literature on smartphone-based ecological momentary assessment of well-being, a systematic review was conducted and reported in accordance with the Preferred Reporting Items for Systematic Review and Meta-Analysis (PRISMA) guidelines (Moher et al. 2009). Titles and abstracts of collected articles were screened for eligibility and were included if (1) (subjective) well-being, happiness, or positive affect/mood was assessed using ecological momentary assessment (EMA was defined as at least two assessments per day for a number of days in natural settings), (2) a smartphone (application) was used, and (3) healthy participants were included (adults or adolescents).

#### Information Source and Search Strategy

In September 2019 and an update in August 2020, the search for relevant articles was conducted in the bibliographic databases PubMed and Web of Science. Additional articles that were missed during this search were identified via reference lists of the selected articles. The search strategy included combinations of search terms related to (1) ecological momentary assessment, (2) well-being/wellbeing and (3) smartphone research (see Table 1). The search applied iterative combinations of these categories by employing the Boolean search operators AND (horizontal) and OR (vertical).

**Table 1:** Search terms, the search applied iterative combinations of these terms by employing the Boolean search operators AND (horizontal) and OR (vertical)

Search term 1	Search term 2	Search term 3
Momentary assessment/measures	(Subjective) well-being	Smartphone
Experience sampling	(Subjective) wellbeing	Smartphone application
Ecologic(al) momentary assessment	Quality of Life	Mobile device
Ambulatory assessment	Satisfaction with Life	iPhone
Ambulatory monitoring	Happiness	Android
Ambulatory measures	Positive affect	
Moment-to-moment measures		

#### Study Selection and Data Extraction

**Fig. 1:**
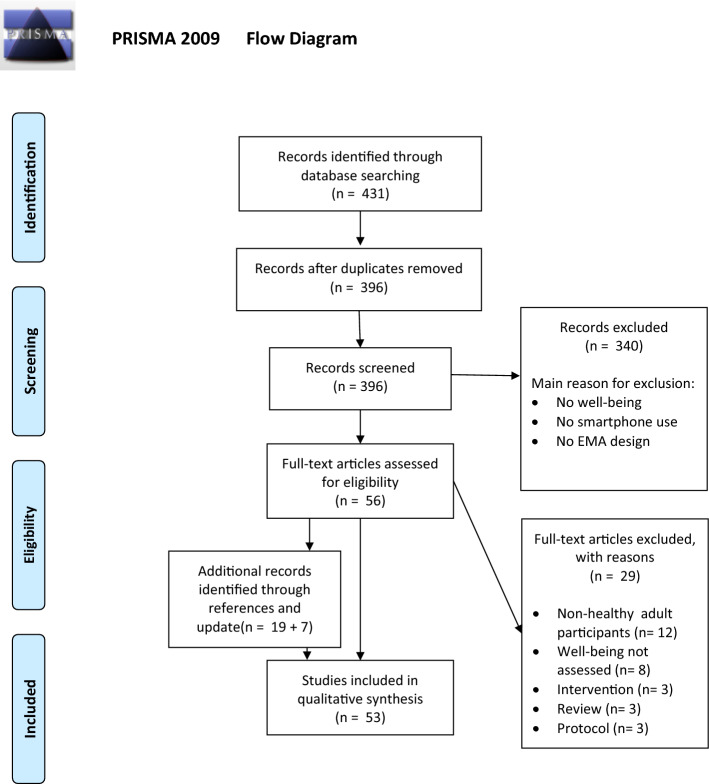
PRISMA Flow Diagram of the included studies

A PRISMA flow diagram of the study selection process is presented in Fig. 1. All identified titles and abstracts were screened for eligibility. In cases of insufficient information to determine eligibility, papers were subjected to further screening. The first author screened the full text reports and decided whether papers met the inclusion criteria. Uncertainties and disagreement were resolved through discussions. Articles were excluded if they met one of the following criteria: (1) no smartphone-based EMA of well-being, (2) non-healthy participants, (3) review papers; (4) descriptive planned studies or methodological papers; or (5) studies that used smartphone applications only for an intervention instead of data collection.

#### Other EMA Data Collection Devices

To put the findings of smartphone-based EMA studies in perspective, we conducted an extra systematic review to EMA studies using other data collection devices, such as palm tops or PDAs with a similar search strategy as described above (see supplementary data Sheet 2 for the keywords, search details and PRISMA Flow Diagram). We compared the designs and results of the two groups of studies.

## Results

### Study Selection and Characteristics

The initial electronic database search resulted in 368 hits in PubMed and 63 hits in Web of Science. When removing duplicates, 396 original articles remained. After scanning the titles and abstracts of those articles based on the selection criteria, 56 articles remained. These articles were examined and read fully. In addition, based on references we included 19 additional articles. After excluding 29 articles based on the full-text reading, 46 articles met our selection criteria and were included in the study. In the update of August 2020, we added 7 new studies, resulting in a total of 53 studies (see Fig. 1).

Tables 2 and 3 provide an overview of the included studies, the samples and the design characteristics (see supplementary file S1 for more detailed characteristics of the reviewed studies). The first smartphone-based EMA study was published in 2010 and the number of studies published increased exponentially over the years, with 38 of the 53 studies published in 2017–2020. Most studies were conducted in Germany (k = 10), the UK (k = 8), the Netherlands (k = 9), USA (k = 8), Australia (k = 5) and South Korea (k = 2). Single studies were conducted in Canada, Israel, China, Japan, and Switzerland. The remaining studies (k = 6) used participant samples from multiple countries.

1. Sampling and measures
  The median sample size of the 53 studies was 97 participants. There was a lot of variability in the sample sizes with a range of 15 participants to 31,302 participants, resulting in an average number of 2212.15 participants (*SD* = 6687.55). A few studies included samples over 1000 participants, e.g. the UK-wide and freely available *Mappiness* app (studies 3, 12, 13, 18, 44 in Table 2) and the worldwide *Track Your Happiness* app (study 1).
  The mean age of the participants was available for 41 studies and ranged from 16.0 to 60.1. Across all studies, the mean age was 30.8 (*SD* = 10.5). The proportion of females included ranged from 5.2 to 100% with a mean of 57.4%. One study included adolescents (36), whereas the rest included adult participants. In 18 studies (34%), participants were only or mostly students. In three studies, participants were employees (17, 34, 38, 48) and two studies included only women (25, 53) (see Table 2 for a full overview of the sample characteristics).
  Most studies (k_number of studies_ = 30, 56.6%) assessed momentary happiness with a single question (variations on “To what extent are you feeling happy?”) using different scales from 0/1 (*not at all*) to 5/7/9/10/100 (*very*/*strongly*). Five studies used the 6-item Short Mood Scale or Multidimensional Mood Questionnaire (valence, calmness and energetic arousal) (2, 5, 9, 32, 38), five studies used positive affect items from the PANAS to rate (e.g. relaxed, excited, energetic) (33, 36, 37, 42, 52), and three a grid of valence (positive/negative) and arousal (8, 11, 19). One study used the Warwick-Edinburgh Mental Well-Being Scale with 14 items (26). Eight studies used other measures, either a qualitative measure (“Select the emotions you have experienced in the last few hours.”) (6, 25, 35) or a combination of other positive mood adjectives (e.g. relaxed, calm) (29, 34, 40, 41, 43).
  The 53 studies measured well-being in different contexts. Momentary well-being in relation to natural environmental variables and contexts (e.g. nature, daylight, urban areas) was assessed in 11 studies (3, 6, 18, 21, 26, 27, 28, 36, 40, 44, 50). Well-being was assessed in relation to physical activity and/or sedentary behavior (k = 9) (2, 5, 9, 19, 25, 38, 43, 48, 53), cognitive processes (e.g. visual search, emotion detection) (30, 31, 32), health (20, 22, 23) alcohol (13, 37, 42), mind wandering (1, 4), food (24, 33), work (12, 17, 51, 52), and sleep (16, 46). Two studies focused on fluctuations of well-being in daily life (34, 45). Single studies measured momentary well-being in the context of homesickness (41), phone use (49), immigrants (14), soccer (10), music (8), fitness exposure (39), transport (47) and social interactions (7). The goal of three studies was to predict momentary mood based on objective data and compared this to EMA data (11, 29, 35). Finally, one study compared different sampling strategies to assess the effect of context on mood (15) (see Table 3).
2. Use of objective data
  Of the 53 studies, 22 studies used only self-reported data, whereas 31 studies (58.5%) included objective, passively measured data.Ten studies used an accelerometer to measure movements (2, 5, 9, 16, 19, 25, 36, 38, 48, 53). Eight studies used GPS location data of the smartphone to investigate well-being in the (natural) environment (3, 12, 13, 15, 18, 26, 44, 45). Four studies used a combination of GPS and accelerometer data (6, 21, 47, 50). One study assessed phone use (49) or the heart rate variability (52). Single studies used an exposimeter of radiofrequency-electromagnetic fields (23) or a light sensor on the clothes of participants to sample the amount of daylight every minute (40). Furthermore, five studies used multiple objective measures, such as a combination of GPS data, time of day, temperature, and neighborhood information (28) or GPS, accelerometer, light, microphone, calls, texts, and Wi-Fi (46). The studies predicting mood used objective measures of mobile phone use (call events, screen use, application use and mobile camera use) (11) or a wide range of (body) sensing data, such as acceleration meter data, heart rate, light level, Wi-Fi signals and GPS location (29, 35).
3. Schedule
  The majority of studies, (k = 43, 81.1%) monitored participants during one specified wave of data collection. The study duration ranged from 1 to 56 uninterrupted days with a mean of 12.8 days (*SD* = 12.8) and median of 7 days. In eight studies the duration was up to the participant (1, 3, 12, 13, 18, 19, 32, 44). Two studies used multiple waves of data collection (7, 28), with 3 times 21 days and 4 times 7 days (each time a different week of the month), respectively.
  The average number of questions per EMA ranged from 1–57, with a mean of 13.1 (*SD* = 11.8). The average completion time, based on the report of 13 studies, was somewhat more than 1.5 min (104.9 s, *SD* = 50.5 s).
4. Technology and administration
  In most studies (k = 33) participants could use their own smartphone in the study, either an Android smartphone (k = 16), iPhone (k = 9) or both (k = 8). In 13 studies, research smartphones with the application installed were provided to the participants. The remaining studies (k = 7) did not specify what type of smartphones or operating systems were used.
  Applications were especially developed to answer the research question and included research specific functions. For example, to measure happiness in relation to food, the SnackImpuls application has the additional feature to quickly categorize and select food choices (33) and the MuPsych app plays music (8). The movisensXS software was used by seven different research teams to create an adapted EMA application (2, 5, 15, 24, 38/48, 40, 52). The (data of the) Mappiness app (k = 5) (3, 12, 13, 18, 44), the Well-being Science app (k = 2) (10, 45), SnackImpuls app (k = 2) (33, 37), the CalFit app (21, 50) and a specific movisensXS app (38, 48) were used in multiple publications to answer different research questions.
  Excluding the double applications, 45 publications used unique applications and 25 of those 45 studies included objective data in addition to self-report to answer the research question. In 13 of the 25 applications (52.0%), the objective data collection (e.g. GPS and accelerometer data) was integrated in the application, using smartphone sensors. In the other studies, either a different application was developed to collect the objective data (k = 2) (6, 11), an additional accelerometer to wear on the arm, chest or hip was provided (k = 8) (2, 5, 9, 25, 36, 38, 40, 53) or an additional exposure meter (23) or heart rate meter (52) was used.
5. Prompting strategy
  The time or interval-contingent design was the most common sampling form (k = 42, 79.2%). Participants received on average 5.0 prompts per day (*SD* = 2.9) on random times on their smartphones, with a range of 2–12 times. Seven studies used an event-contingent design. In three of those studies the participant had to initiate the EMA after eating (24), a trip (47) or a social interaction (7, 41). In other studies participants were prompted with a questionnaire when an accelerometer measured physical activity that surpassed or fell below a predefined activity threshold (9), when they started to listen to music (8) or based on the exposimeter data (23).
  Three studies used a mixed design (28, 38, 8), using a combination of time and event-contingent sampling based on physical activity or location. A mixed design was used to prevent the collection of too little data when not enough events occurred. Lastly, one study (15) compared four different sampling designs: based on time, based on combined time and distance (prompt when moving), based on location (prompt when moving to a new location) and, lastly based on land use and population density (prompt when moving to a location with a different type of land use or with a different population density). Location-based sampling resulted in less prompts compared to time-based sampling, but in more triggers for unique locations and a greater spatial spread.
  In 41 of the 53 studies (77.4%), participants were prompted with a buzz or auditory signal of the smartphone application. Three studies used text messages to remind the participants to open the app and answer questions (10, 22, 45) and one study used a smartwatch to signal participants (29). In the remaining six studies, the participants had to initiate the questionnaire themselves. Participants had to start a questionnaire after a predefined event in five event-based sampling studies (7, 8, 24, 41, 47). In the other two studies, participants had to initiate an EMA in the morning and the evening (21, 50) or somewhere in three predefined timeslots (31).
6. Response and Compliance
  Only 25 (47.2%) studies reported information on the compliance with the EMA design. Additionally, seven studies provided enough information to calculate the compliance rate. Based on the 32 studies, participants completed on average 71.6% (*SD* = 14.1%) of all EMAs with a range of 43–95%.
  Five studies reported a relation between compliance and another participant or study variable. Higher compliance was found in the first three days compared to the fourth until seventh day (37) and during weekends compared to weekdays (42). In study 25, no differences in compliance levels for assessments over the day has been found, whereas in study 20 surveys were most often responded to between 12 and 2 pm. Also, participants were found to be most often at home, alone, or involved in work/study when responding (20). In study 42, EMA compliance was unrelated to age, gender and other personal characteristics.
  Based on 9 of the 13 studies with a research phone that did report the compliance, the average completion rate is 76.4% (SD = 0.08). This is not significantly different from studies where participants could use their own smartphone (70.6%, SD = 0.16, based on 23 studies), *p* = 0.294.
  The compliance levels are not different in studies when participants received incentives (k = 14, compliance based on reports of 9 studies: 72.1%) compared to when participants did not receive incentives (k = 30, compliance based on 19 studies: 69%), *p* = 0.614. Participants in the remaining 9 studies received course credit, participated in a lottery or received a gift (compliance based on 4 reports: 82%). This compliance is also not different from the compliance in the paid or non-paid studies (*p* = 0.168 and *p* = 0.127).
  Studies with the highest compliance rates (> 84%) seem to last either 7 or 14 days with 3–6 prompts per day. Furthermore, studies with a relatively long duration tend to have lower compliance, as 30 days sampling (22) resulted in a compliance of 50% and 21 uninterrupted days of sampling in a rate of 43% (42). However, based on reports of 27 studies, there is no significant correlation between the reported compliance rates and the duration of the study (*r* = − 0.144, *p* = 0.458) or the prompts per day (*r* = 0.243, *p* = 0.204).
  Besides overall compliance, the timing of responses to prompts is important in EMA studies. This is essential information, since the validity of EMA studies is based on momentary experiences, i.e. the answering of the questions should be in the moment. Whereas a number of studies reported a limited time (90 s–1 h) to respond to a prompt, only three studies actually reported the average latency time between the prompt and answer. The assessments were completed within 38 min (51), 11.6 min (22) or within 28 s (23). In addition, study 20 reported that 66.5% (n = 260) of the EMA’s was responded to within a minute, only 2 took more than 5 min.
  Most studies (k = 43) recruited a target sample, whereas in 10 studies anonymous participants downloaded a freely available and widely advertised application (1, 3, 12, 13, 18, 19, 22, 26, 32, 44). Both methods of recruiting lead to participant drop out during the study, resulting in a difference between the number of participants in the initial enrollment sample and analytical sample. Thirty studies reported this attrition rate and the average dropout rate was 17.1% (*SD* = 20.8%) with a range of 0–96.8%. Based on reports of 25 and 32 studies respectively, the attrition rate was not related to study duration (r = 0.106, *p* = 0.550) or the number of prompts per day (r = 0.233, *p* = 0.241).
7. Analyses
  Most studies (k = 40, 75.5%) analyzed the data using multilevel models or mixed models (fixed and random effects), taking into account the nested nature of EMA data. EMA data consists of repeated measurements of participants over multiple days. Therefore, in the analysis, adjusting for individual effects is necessary. However, five studies only looked at group differences and did not analyze differences on the individual level (10, 14, 24, 30, 31). In addition, two studies computed the correlation between well-being and the other measure separately for every participant and averaged these correlations (5, 8). One study analyzed the data at response level instead of participant level (20). Finally, two studies did not perform any data analysis (6, 15) and three studies only provided a summary and descriptive data to show the feasibility of smartphone-base EMA (17, 23, 34).
8. Results of the studies
  Table 4 describes the results of the reviewed studies, grouped by context, with examples of statistical results of interest. To briefly summarize, momentary well-being fluctuated daily and weekly, with on average higher well-being in the evening and weekend. Yet, often both daily and weekly fluctuations disappear when type of place, physical activity or other environmental variables are controlled for. On average, being in a natural environment in daily life (e.g. walking in the park) and physical activity over the day is associated with positive affect and higher well-being. Working was ranked lowest in happiness levels, but employees do show fluctuations based on the task, where you work (office, home or somewhere else), whether you are alone or with others; the time of day or night working and personal characteristics (e.g. lower WB associated with working when married, but higher when having children). Work stress is negatively related to positive affect.
  The effects of mind wandering on positive affect are inconclusive with a negative and no relation. Eating is related to positive affect, with stronger effects for vegetable consumption and snacks. Drinking alcohol is related to higher momentary positive affect on average, but this increased well-being does not last or spill to other moments. Sleep and well-being were related, with a stronger effect of sleep on mood than of mood on sleep quality. Furthermore, on average, positive affect was associated with faster visual search reaction times, but not with other cognitive measures. Focusing attention on well-being by completing multiple questionnaires about well-being for a few weeks does increase well-being. One study investigated why internal migrants report lower levels of happiness than locals, even after accounting for socio-economic factors. EMA showed that migrants spend less time to happiness-producing activities such as active leisure, and social parties than locals. Furthermore, single studies show that exposure to fitness inspiration online and social media use is related to lower happiness, whereas listening to music and watching soccer is related to higher momentary happiness on average. Walking and cycling is better for your mood than sitting in a bus or auto. Lastly, the studies predicting mood and well-being based on objective data, such as phone use, were successful in 55% to 76% of their mood predictions. However, other models, for example based on the mean mood state, performed better than the personalized models using objective data. See Table 4 and the supplementary data for more detailed results and the corresponding studies.
9. Limitations and risk of bias

**Table 2:** Characteristics of the samples included in the 53 reviewed studies

Nr	Study	Study location	Sample N	Sample	Mean age	SD age	% female
[1]	Killingsworth and Gilbert (2010)	USA	2.250	Population	34		41.2
[2]	Bossmann et al. (2013)	Germany	62	Students	21.4	1.8	14.5
[3]	MacKerron and Mourato (2013)	UK	21.947	Population	N/S		45
[4]	Poerio et al. (2013)	UK	24	Convenience	24.17	2.9	45.8
[5]	Von Haaren et al. (2013)	Germany	29	Students	21.3	1.7	N/S
[6]	Doherty et al. (2014)	Canada	15	Park visitors	20–45		33.3
[7]	Ram et al. (2014)	USA	136	Stratified	47.64	18.85	51
[8]	Randall et al. (2014)	Australia	327	Community /students	21.02	6.18	76.1
[9]	Kanning et al. (2015)	Germany	69	Random	60.1	7.1	49
[10]	Stieger et al. (2015)	Germany	213	Convenience	24.5	8.4	58
[11]	Asselbergs et al. (2016)	Netherlands	27	Students	18–25		78
[12]	Bryson and MacKerron (2016)	UK	26.700	Population	N/S		44.4
[13]	Geiger and MacKerron (2016)	UK	31.302	Population	35.1		N/S
[14]	Hendriks et al. (2016)	Germany	150	Students/Convenience	21.7	3.1	82
[15]	Törnros et al. (2016)	Germany	143	–	18		57.3
[16]	DeMasi et al. (2017)	USA	53	Students	19.83	1.99	55.3
[17]	Engelen et al. (2017)	Australia	22	Population	26–45		50
[18]	Fujiwara et al. (2017)	UK	N/S	Population	N/S		47
[19]	Lathia et al. (2017)	UK	12.838	Population	N/S		43
[20]	Liddle et al. (2017)	Australia	40	Convenience	23	3.15	80
[21]	Triguero-Mas et al. (2017)	Spain/UK /Netherlands /Lithuania	406	Cluster	51	26	53.2
[22]	Van der Krieke et al. (2017)	Netherlands	629	Population	49.9	13	82
[23]	van Wel et al. (2017)	Netherlands	34	Online	32		56
[24]	Wahl et al. (2017)	Germany	38	Students	24.47	5.88	73.7
[25]	Zenk et al. (2017)	USA	97	Specific	25–65		100
[26]	Bakolis et al. (2018)	UK	108	Population	31.1	11.1	75
[27]	Beute and de Kort (2018)	Netherlands	57	Local database	33	13.7	66.1
[28]	Birenboim (2018)	Israel	91	Students	26.01	3.13	59.8
[29]	Gloor et al. (2018)	Germany/USA	17	Voluntary	23–56		70
[30]	Ludwigs et al. (2018)	Germany	90	Students	21.9/21.4	4.6/4.3	83.7/84.2
[31]	Maekawa et al. (2018)	Japan	33	N/S	20–35		24.2
[32]	von Stumm (2018)	Multiple	770	Population	35.38	1.29	31
[33]	Wouters et al. (2018)	Netherlands	269	Convenience	35	8.91	73
[34]	Yang et al. (2018)	South Korea	97	Convenience	37.7	10.8	5.2
[35]	Zhang et al. (2018)	China	30	Students	18–30		57
[36]	Bejarano et al. (2019)	USA	26	Community	15.96	1.56	42.3
[37]	Duif et al. (2019)	Netherlands	162	Community	36.07	9.23	67.2
[38]	Giurgiu et al. (2019)	Australia/ Germany	86	Employees	33.7	9.3	62.8
[39]	Griffiths and Stefanovski (2019)	Australia	108	Students	19.47	2.89	78.7
[40]	Itzhacki et al. (2019)	Netherlands	25	Convenience	23.7	3.8	51.9
[41]	Nauta et al. (2019)	Netherlands	82	Students	20	1.8/1.2	67
[42]	O'Donnell et al. (2019)	Australia	83	Community	21.42	3.09	75.9
[43]	Schultchen et al. (2019)	Germany	51	Community	23.50	2.6	21.6
[44]	Seresinhe et al. (2019)	UK	15.444	Population	N/S		48
[45]	Stieger and Reips (2019)	Germany	213	Community	24.5	8.4	58
[46]	Triantafillou et al. (2019)	USA	206	Online	39.30	10.3	80.8
[47]	Glasgow et al. (2019)	USA	229	Online	32.67	10.5	55.1
[48]	Giurgiu et al. (2020)	Australia/Germany	80	Employees	33.9	9.5	65
[49]	Johannes et al. (2020)	Netherlands	75	Phone users	21.9	2.5	70.7
[50]	Kondo et al. (2020)	Spain/UK/Netherlands /Lithuania	368	Cluster	51	33.6	52
[51]	Ryu et al. (2020)	South Korea	89	Convenience	37.2	10.5	5.6
[52]	Schilling et al. (2020)	Switzerland	201	Convenience	38.6	10.1	35.8
[53]	Yang et al. (2020)	USA	185	Cohort study	41.03	5.86	100

**Table 3:** Design characteristics of the 53 included Ecological Momentary Assessment studies of well-being

Nr	Study	Sample size	Context of assessment	WB measure	Study days	Times /day	Nr of items	EMA design	Phone	Application name	Objective data	Obj in app	Compliance % *
[1]	Killingsworth and Gilbert (2010)	2.250	Mind wandering	Feeling good (0–100)	N/S	3	3	Time	iPhone	Track Your Happiness	–	–	83%
[2]	Bossmann et al. (2013)	62	Physical activity	Valence, calmness and arousal	7	Every hour	6	Time	Research phone	MovisensXS	Accelerometer		
[3]	MacKerron and Mourato (2013)	21.947	Natural environment	Happy (0–100)	N/S	2	7	Time	iPhone	Mappiness	GPS	Yes	48%
[4]	Poerio et al. (2013)	24	Mind wandering	Happy (1–5)	7	12	7	Time	Research phone	ESAMO	–	–	88.4%/ 79.3%
[5]	Von Haaren et al. (2013)	29	Physical activity	Valence, calmness and arousal	2	Every 2 h	6	Time	Research phone	MovisensXS	Accelerometer		
[6]	Doherty et al. (2014)	15	Nature	Select emotion	1	Every 35 min	14	Time	Research phone	2 Java programs	GPS + accelerometer	No	68%
[7]	Ram et al. (2014)	136	Social interactions	Happy (1–100)	3 × 21	N/A	27	Event	Research phone	N/A	–	–	
[8]	Randall et al. (2014)	327	Music	Grid: Valence and arousal	14	2	4	Event	iPhone	MuPsych	–	–	
[9]	Kanning et al. (2015)	69	Physical activity	Valence, calmness and arousal	3	N/A	6	Event	Research phone	MyExperience	Accelerometer	No	
[10]	Stieger et al. (2015)	213	Soccer	Well-being (0–100)	14	3	1	Time	Android	Well-being Science App	–	–	94%*
[11]	Asselbergs et al. (2016)	27	Predicting mood	Grid: Valence, arousal (1–10)	42	5	2	Time	Android + iPhone	eMate/ iYouVU	Multiple	No	77%*
[12]	Bryson and MacKerron (2016)	26.700	Work	Happy (0–100)	N/S	2	7	Time	iPhone	Mappiness	GPS	Yes	
[13]	Geiger and MacKerron (2016)	31.302	Alcohol	Happy (0–100)	N/S	2	7	Time	iPhone	Mappiness	GPS	Yes	48%
[14]	Hendriks et al. (2016)	150	Immigrants	Happy (1–11)	14	6	4	Time	Android + iPhone	Happiness analyzer	–	–	91%
[15]	Törnros et al. (2016)	143	Sampling strategies	N/S	7		N/S	Multiple	N/S	MovisensXS	GPS	Yes	
[16]	DeMasi et al. (2017)	53	Sleep	Happy (1–9)	56	4	2	Time	Android	Funf Open Sensing	Accelerometer	Yes	
[17]	Engelen et al. (2017)	22	Work	Happy (1–10)	5	4	13	Time	N/S	LifeData RealLifeExp	–	–	58%
[18]	Fujiwara et al. (2017)	N/S	Airport noise	Happy (0–100)	N/S	3	7	Time	iPhone	Mappiness	GPS	Yes	
[19]	Lathia et al. (2017)	12.838	Physical activity	Grid: Valence and arousal	N/S	2	5	Time	N/S	Mood-tracking	Accelerometer	Yes	
[20]	Liddle et al. (2017)	40	Quality of life	Feeling good (1–7)	7	6–8	5	Time	Android	My life tracker	–	–	
[21]	Triguero-Mas et al. (2017)	406	Natural environment	Happy person	7	2	29	Time	Android	CalFit	GPS + accelerometer	Yes	
[22]	Van der Krieke et al. (2017)	629	Mental health	Feeling good (0–100)	30	3	43	Time	Android + iPhone	HND	–	–	50%*
[23]	van Wel et al. (2017)	34	Health	Well-being (1–6)	2	N/A	11	Event	Android	ExpoMDiary	Exposimeter	No	74%
[24]	Wahl et al. (2017)	38	Food	Eating happiness	8	N/A	6	Event	N/S	MovisensXS	–	–	75%
[25]	Zenk et al. (2017)	97	Physical activity	Select emotion	7	5	19	Time	Research phone	N/S	Accelerometer	No	70%
[26]	Bakolis et al. (2018)	108	Environment	Mental Well-Being Scale	7	7	20	Time	Android + iPhone	Urban Mind	GPS	Yes	61%*
[27]	Beute and de Kort (2018)	57	Nature and daylight	Happy (1–7)	6	8	45	Time	Research phone	N/S	–	–	80%
[28]	Birenboim (2018)	91	Environment	Happy (1–7)	4 × 7	4	21	Mixed	N/S	N/S	Multiple	Yes	52%*
[29]	Gloor et al. (2018)	17	Predicting emotions	Pleasance and activation	49	4–7	3	Time	N/S	Happimeter	Multiple	Yes	
[30]	Ludwigs et al. (2018)	90	Attention to WB	SWB (0–10)	14	6	6	Time	Android	N/S	–	–	
[31]	Maekawa et al. (2018)	33	Visual processes	Happiness (0–10)	14	3	8	Time	Android + iPhone	Visual Search	–	–	95%*
[32]	von Stumm (2018)	770	Cognitive capacities	Valence, calmness and arousal	N/S	1–4	57	Time	iPhone	moo-Q	–	–	
[33]	Wouters et al. (2018)	269	Food	PANAS	7	10	12	Time	Android	SnackImpuls	–	–	69%
[34]	Yang et al. (2018)	97	Fluctuations	Positive affect	7	4	25	Time	Android	N/S	–	–	60%
[35]	Zhang et al. (2018)	30	Predicting Emotions	Select emotion	30	3	6	Time	Android	MoodExplorer	Multiple	Yes	
[36]	Bejarano et al. (2019)	26	Environment and activity	5 items PANAS (1–5)	20	4	16	Time	Research phone	PETE App	Accelerometer	No	88%
[37]	Duif et al. (2019)	162	Alcohol	PANAS	7	10	12	Time	Android + iPhone	SnackImpuls	–	–	72%
[38]	Giurgiu et al. (2019)	86	Physical activity and sedentary	Valence, calmness and arousal	5	10	6	Mixed	Research phone	MovisensXS	Accelerometer	No	76%
[39]	Griffiths and Stefanovski (2019)	108	Exposure to fitness inspiration	Happy (0–100)	7	6	10	Time	Android + iPhone	Life Data	–	–	78%
[40]	Itzhacki et al. (2019)	25	Light and time of day	Relaxed, calm, energetic, happy,	7	9	22	Time	Research phone	MovisensXS	Light	No	65%*
[41]	Nauta et al. (2019)	82	Homesickness	Happy, pleased, joyful, enjoyment	14	N/A	10	Event	N/S	TEMPEST app	–	–	
[42]	O'Donnell et al. (2019)	83	Alcohol use	PANAS: e.g. happy (0–5)	21	3	13	Time	iPhone	–	–	–	43%
[43]	Schultchen et al. (2019)	51	Physical activity and eating	Positive adjectives (0–100)	7	6	17	Time	Android + iPhone	PsyDiary app	–	–	84%
[44]	Seresinhe et al. (2019)	15.444	Environment	Happy (0–100)	N/S	2	7	Time	iPhone	Mappiness	GPS	Yes	
[45]	Stieger and Reips (2019)	213	Fluctuations	Well-being (0–100)	14	3	1	Time	Android	Well-being Science App	GPS	Yes	87%
[46]	Triantafillou et al. (2019)	206	Sleep	Mood (0–8)	42	2	6	Time	Android	Purple Robot	Multiple	yes	
[47]	Glasgow et al. (2019)	229	Transport	Mood: happy, cheerful, optimistic, pleased, interested	7	N/A		Event	Android	Daynamica	GPS + accelerometer	Yes	
[48]	Giurgiu et al. (2020)	80	Physical activity and sedentary	Valence, calmness and arousal	5	10	6	Mixed	Research phone	MovisensXS	Accelerometer	No	77%
[49]	Johannes et al. (2020)	75	Online vigilance	Happy	5	8	18	Time	Android	PACO	Phone use	Yes	60%
[50]	Kondo et al. (2020)	368	Natural environment	Happy	7	2	29	Time	Android	CalFit	GPS + accelerometer	Yes	
[51]	Ryu et al. (2020)	89	Work stress	Positive affect	7	4	25	Time	Android	N/S	–	–	64.7%
[52]	Schilling et al. (2020	201	Work stress	Positive affect	2	8	14	Time	Research phone	MovisensXS	heart rate variability	No	80.9%
[53]	Yang et al. (2020)	185	physical activity	Happy	7	8	N/S	Time	Android	N/S	Accelerometer	No	80.3%

N/A: not applicable, N/S: not specified

The compliance rate was not reported in 7 studies, but could be computed using other data in the publication (indicated by *)

See the supplementary table for more characteristics

**Table 4:** Summary of the results of the 53 reviewed studies and examples of estimated effects of interest on mood or well-being, sorted by study context

	Nr	Study	Results related to mood or well-being	Estimated effect on WB (SE)
Fluctuations	[34]	Yang et al. (2018)	Methodological: A smartphone application is feasible to measure the fluctuations of well-being	
	[45]	Stieger and Reips (2019)	On average, WB fluctuates daily (low in the morning, high in the evening) and over the week (low just before the beginning of the week, highest near the end of the week). Being on deviating altitudes from normal relates to higher WB (might be explained by leisure time). Weather conditions have no significant effect on WB	Hour of the day: b = 0.64* (.08) Day of the week: b = 0.40* (0.18) Altitude: 0.01* (0.01) Rainfall: − 0.33 (.36), wind speed: − 0.34 (0.27), temperature: 1.10 (0.80)
Natural environment	[3]	MacKerron and Mourato (2013)	Participants are happier outdoors in all green or natural habitat types (e.g. in mountains or heathland) than they are in urban environments Weather did influence WB, higher when sunny, lower when raining	Outdoors: FE = 2.32* (.45) Mountains/heathland: FE = 2.71* (.87) Walking, hiking: FE = 2.55* (.18) Rain: FE = − 1.37* (.22), Sun: FE = 0.46* (.18)
	[6]	Doherty et al. (2014)	Methodological: The combination of passive and interactive techniques can enhance the ability to understand how contact with nature enhances health and well-being	
	[18]	Fujiwara et al. (2017)	Being near an airport is not associated with happiness, whereas being in areas with aircraft noise (depending on the dB) is associated with lower levels of happiness and relaxation	Being near an airport: FE = 0.069 (0.45) Aircraft noise: FE = − 6.344* (3.039)
	[21]	Triguero-Mas et al. (2017)	More contact with natural outdoor environments is related to better well-being	Contact with surrounding green: females: b = 4.01* (0.77, 7.24), males: b = 3.38 (− 0.15–6.90)
	[26]	Bakolis et al. (2018)	Being outdoors, exposure to natural features including trees, the sky, and birdsong has a beneficial impact on momentary mental well-being	Being outdoors (MD: 2.90, *p* < .001*), seeing trees (MD: 1.31, *p* < .001*), hearing birds singing (3.71, *p* < .001*), seeing the sky (1.49, *p* < .05*), and feeling in contact with nature (3.51, *p* < .001*)
	[27]	Beute and de Kort (2018)	Higher levels of nature and daylight are related to more positive affective states, whereas time of the day is not significantly associated with positive affect	Nature: B = .025* (.009) Daylight: B = .027* (.009) Time of the day: B = .033 (.017)
	[28]	Birenboim (2018)	Happiness is influenced by time of the day and day of the week and situational variables and environmental characteristics including type of activity and environment, place characteristics, and company	Activity type: adj R^2^: 0.255* Company: adj R^2^: 0.214* Place type: adj R^2^: 0.234* Neighborhood: adj R^2^: 0.218* Temperature: adj R^2^: 0.196* Clouds: adj R^2^: 0.234 Time of day: adj R^2^: 0.203* Weekend: adj R^2^: 0.209*
	[36]	Bejarano et al. (2019)	Being at home was negatively related to positive affect, while being at school, in the car, or outdoors was positively associated with positive affect. Too hot or cold weather was negatively related to positive affect	At home (β = − 1.47*), At school (β = 1.19*), In the car (β = 0.77*), Outdoors (β = 1.66*) Proximity to vegetation (β = 0.21*) Weather just right (β = 0.66*). Too hot (β = − 0.80*) or too cold (β = − 0.53*) weather
	[40]	Itzhacki et al. (2019)	Time of day significantly modulated positive mood, peaking around 2 pm. Variability in light intensity did not significantly affect positive mood	Time of day: *p* = .0012* Variability in light intensity: *p* = .11 Average light intensity: *p* = .31
	[44]	Seresinhe et al. (2019)	People do report themselves to be happier in a more scenic location and in a more natural habitat and rural environment	Scenicness: b = 2.77*, *p* < .001 Natural habitat: b = 0.57*, *p* < .001 Rural: b = 0.61*, *p* < .001
	[50]	Kondo et al. (2020)	There was a positive relationship of exposure to natural outdoor environments and positive affect	OR: 1.39*, 95% CI: 1.06, 1.81
Physical activity	[2]	Bossmann et al. (2013)	Energetic arousal and valence were positively influenced by the intensity of the physical activity 10-min prior to the assessment. On average, as activity increased, positive feelings and arousal increased	Physical activity: b = 0.003, std effect: 0.07, *p* = .005*
	[5]	Von Haaren et al. (2013)	No relation between activity and affect in inactive people. Inactive people hardly show any active episodes during the day. Therefore, it is difficult to analyze the relation between affect and PA of inactive people	86% of the time in sedentary state Mean MET: r = − 0.03, *p* = .45 Minutes light activity: r = 0.08, *p* = .65
	[9]	Kanning et al. (2015)	When older individuals were more physically active, they felt more energized and agitated, but did not show better mood (valence)	Activity on valence: FE = < .001, *p* = .86 Activity on energy: FE = .0017*, *p* = .02 Activity on calmness: FE = -.0017*, *p* < .001
	[19]	Lathia et al. (2017)	The frequency with which people physically move daily (both self-report and accelerometer data) is related to physical health and happiness. Individuals who are physically more active are happier, both in general and in the moment	Self-report activity: r = .08*, *p* < .001, d = .16, Sensed activity (accelerometer): r = .03*, *p* = .002, d = .06
	[25]	Zenk et al. (2017)	Daily positive affects was not related to physical activity (PA) or sedentary behavior. More PA during the day was associated with increased subsequent positive affect, while more sedentary behavior during the day was associated with reduced positive affect	WB—daily activity (minute): b = 0.14 (0.1) WB—daily sedentary: b = − 14.68 (8.9) Daily activity on WB: OR: 1.02* Daily sedentary on WB: OR: 0.99*
	[38]	Giurgiu et al. (2019)	Momentary sedentary time, but not physical activity had a negative effect on valence. Time of the day is significantly associated with mood, but day of the week not	Sedentary time: β = − 0.082, *p* < .001* Physical activity: β = − 0.037, *p* = .074 Time: β = − 0.212, *p* < .001* Day (weekend): β = .054, *p* = .059
	[43]	Schultchen et al. (2019)	Positive affect was related to higher subsequent physical activity and vice versa, physical activity was related to higher subsequent positive affect	Affect to physical activity: b = .067, SE = .029, *p* = .027* Physical activity to affect: b = .193, SE = .029, *p* < .001 *
	[48]	Giurgiu et al. (2020)	Mood did not significantly predict sedentary time between subjects, but sedentary time was negatively predicted by valence and energetic arousal and positively by calmness within subjects	Valence: − 0.03* (0.02) Arousal: − 0.06* (0.01) Calmness: 0.07* (0.02)
	[53]	Yang et al. (2020)	When mothers experienced higher-than-usual positive affect, they engaged in more sedentary time in the same 45-min window	
Work	[12]	Bryson and MacKerron (2016)	Doing paid work is ranked low in terms of happiness. However, well-being at work varies significantly with where you work (higher when at home); what you are doing at the same time (e.g. listening to music); whether you are alone or with others (higher WB when with colleagues or family/friends); the time of day or night you are working (lower WB when working in weekend and after 6 pm); and your personal characteristics (lower WB when working when married, somewhat higher WB when working and having children)	Working: FE = − 5.43* Extra negative effects → + working in weekend: − 2.37 + working after 6 pm: − 2.59 Where: home vs at work: − 4.09 With whom: colleagues (+ 0.64, spouse, partner (+ 5.91), clients (+ 0.72) Combined with music (+ 3.38), admin (− 3.64), eating (+ 2.25)
	[17]	Engelen et al. (2017)	The app was able to measure temporal variance and patterns of mood during work. People vary in their mood during a working day	
	[51]	Ryu et al. (2020)	Momentary PA was negatively correlated with occupational stress in police officers. Work overload and social isolation were positively associated with PA. Work discontent, social tension, and pressure to perform were negatively associated with momentary PA	Occ stress: − .276* (.009) .25 (.14), .48 (.10), 0.45 (.14), − .79 (.16) and − .29 (.14)
	[52]	Schilling et al. (2020)	Positive affect was negatively related to feelings of stress, but not related to cardiorespiratory fitness	Stress: − .34*
Mind wandering	[1]	Killingsworth and Gilbert (2010)	People were less happy when their minds were wandering than when they were not. What people were thinking (10.8% variance explained) was a better predictor of their happiness than what they were doing (4.6% explained)	Mind wandering: slope (b) = –8.79, *p* < 0.001*
	[4]	Poerio et al. (2013)	While sadness tended to precede mind-wandering, mind-wandering itself was not associated with later mood and only predicted feeling worse if its content was negative	Mind wandering on sadness: t = − .29, B = − 0.02 (0.06), *p* = .771 Sad mind-wandering on WB: t = 5.08, B = .27, (0.05), *p* < .001*
Food	[24]	Wahl et al. (2017)	Vegetables consumption influenced eating happiness, sweets on average provided comparable induced happiness to “healthy” food and dinner elicited comparable happiness to snacking	Fruits and vegetables accounted for 24% of total eating happiness score Happiness: dinner (M = 81.47, SD = 14.73), and snacks (M = 79.45, SD = 14.94) Snacks vs lunch (t = − 4.44, *p* = .001, d = − 0.38*) and breakfast, (t = -3.78, *p* = .001, d = − 0.33*)
	[33]	Wouters et al. (2018)	Men and young adults (20–30) significantly increased their food intake after experiencing positive affect, whereas no associations were found in women nor in the other age groups	Energy intake from snacks: β (S.E.) = .01 (.01), *p* = .29 + age: − .24 (.08), *p* < .01* Interaction gender: − .17 (.07), *p* = .02* Males: β (S.E.) = .07 (.03), *p* = .01*
Alcohol	[13]	Geiger and MacKerron (2016)	People are happier at the moment of drinking alcohol, but the overspills to other moments are small. Younger people are happier when drinking compared to older people. Changing drinking levels over time is not related to changing life satisfaction	Alcohol: FE = 3.65, *p* < .001* Happiness of younger people (+ 7.3 points) vs oldest group (+ 3.0 points)
	[37]	Duif et al. (2019)	Within-person momentary positive affect was positively associated with likelihood of next-moment alcohol consumption but not with the quantity of alcohol use. Between persons, levels of momentary affect were not related to alcohol drinking	Within: Positive affect and likelihood alcohol use: OR = 1.21, *p* = 0.01* Within: Positive affect and alcohol use quantity: B = .02, *p* = 0.82 Between persons: likelihood: OR = 0.96, *p* = .62 and quantity: B = 0.07, *p* = .52
	[42]	O'Donnell et al. (2019)	Being happy is correlated to the number of drinks and whether someone drinks. However, alcohol consumption does not predict happiness	Happy and number of drinks: r = 0.09* Happy and drink yes/no: r = 0.09* Alcohol consumption on happiness: B = 0.31, *p* = 0.11
Sleep	[16]	DeMasi et al. (2017)	. Daily physical activity and sleep duration measured with smartphones were positively correlated with and predictive of mood. Nighttime stillness, day of the week and study was not associated with mood	Day of study: FE = − 0.059, *p* = .82 Day of week: FE = 0.04, *p* = .60 Sleep duration: FE = 0.072, *p* = .02* Daytime activity: FE = 0.097, *p* = .004* Nighttime stillness: FE = 0.04, *p* = .13
	[46]	Triantafillou et al. (2019)	The effect of sleep quality and mood is bidirectional, but the effect of sleep quality on mood was larger than the effect of mood on sleep quality	Sleep on mood: FE = 0.344 (0.009), *p* < .001* Mood on sleep quality: FE = 0.132 (0.019), *p* < .001*
Cognitive processes	[30]	Ludwigs et al. (2018)	Paying more attention to one’s subjective well-being (SWB) does increase SWB in general on several questionnaires	Increase in happiness: Happiness Core: (*p* = .08), Life Satisfaction (*p* = .41), Domain Evaluation Questionnaire (*p* = .008*), Flourishing scale (*p* = .036*), satisfaction with life scale (*p* = .004*)
	[31]	Maekawa et al. (2018)	Pop-out search times were unaffected by mood, serial search times were significantly faster for high happiness levels than low happiness levels. Happiness did not influence motor response speed	Happiness on serial visual search times: β = − 96.56, t = − 2.18, *p* = 0.03* Motor response F(1, 32) = 0.37, *p* = 0.55
	[32]	von Stumm (2018)	Short-term and working memory tasks were not associated with positive affect. Having slept enough, being alone and at work was associated with improved cognitive function	Mood and short-term memory (β = .10, SE = .05, t = 2.09, *p* = .037) Mood and working memory (β = 07, SE = .04, t = 1.87, *p* = .061)
Health	[22]	Van der Krieke et al. (2017)	Somatic symptoms are negatively associated with quality of life, but there is significant heterogeneity in this association between persons	Somatic symptoms on QoL: B = − 0.25; *p* < .001* Heterogeneity in the within-person association (variance, 0.02; *p* < .001*)
	[23]	van Wel et al. (2017)	Radiofrequency-electromagnetic fields exposure had minimal influence on well-being	No statistical report
Quality of Life	[20]	Liddle et al. (2017)	High momentary quality of life was significantly related to high occupational enjoyment, being in the presence of someone, being home and having an excellent health status	Occupational enjoyment: β = 4.480 (0.634), *p* < .001* Home: β = 0.815 (0.379), *p* = .03* Social context: β = 0.662 (0.266), *p* = .013* Excellent health: β = 0.768 (0.327), *p* = .019*
Fitness inspiration	[39]	Griffiths and Stefanovski (2019)	Thinspiration and fitspiration were uniquely and interactively associated with lower body satisfaction, higher negative affect, and lower positive affect	Positive affect: Thinspiration: b = − 6.36, *p* < .001, d = − 0.09.* Fitspiration: b = − 2.08, *p* = .022, d = − 0.04* Dual exposure: b = − 5.82, p,.001, d = − 0.08*
Homesick- ness	[41]	Nauta et al. (2019)	Participants felt most homesick when interacting with their parents or using video chat and feeling homesick is associated with more unpleasant and less pleasant affect	Momentary homesickness: β = − .09, F = 8.51, *p* = .004*
Social interaction	[7]	Ram et al. (2014)	Interactions with family members and weekend days boosted happiness	Interactions with family members and happiness: + 1.16 units (*p* < .01*) Weekend days: + 1.88 units (*p* < .01*)
Phone use	[49]	Johannes et al. (2020)	Small relations between online vigilance and well-being. More social apps time is related to worse mood	
Music	[8]	Randall et al. (2014)	Music listening elicited a significant increase in valence and mood over a 3-min listening period. This positive shift in valence was amplified when the initial mood was negative	Music on mood: t(326) = 3.94, *p* < .001, d = 0.22* When mood was negative: t(304) = 13.39, *p* < .001, d = 0.65*
Soccer	[10]	Stieger et al. (2015)	Well-being was higher among soccer spectators than non-spectators during the world cup, with effects increasing as a function of goal difference	Soccer win: t = 2.88, *p* < .01, d = 0.64*
Immigrants	[14]	Hendriks et al. (2016)	Internal migrants distribute less time to happiness-producing activities such as active leisure, social drinking/parties, and activities outside home/work than locals. Internal migrants feel less happy than locals when spending time with friends and while eating	Mean difference happiness locals and migrants: 0.64, F = 4.19, *p* < .05, np^2^ = 0.03.* Diff time spent per day locals and migrant to: Social drinks (F = 7.66, *p* < .01*), Active leisure (F = 4.70, *p* < .05*) Diff happiness when: With friends (F = 4.43, *p* < .05*), eating (F = 3.15, *p* < .10)
Transport	[47]	Glasgow et al. (2019)	Mood was more positive when individuals walked and bicycled versus when they used a bus or automobile. Paths near green space and water were related to positive mood	Walking: 0.20(0.08)*, Cycling: 0.30(0.14)*, Talking with others: 0.26(0.08)**, Green: 0.002(0.01)*
Predicting mood	[11]	Asselbergs et al. (2016)	Mobile phone-based unobtrusive EMA is a technically feasible and potentially powerful method to predict mood based on the data. However, the predictive performance of the personalized predictive regression models was inferior to that of naive benchmark prediction models (mean model, predicted the mood to be equal to the average observed mood) that are agnostic of mobile phone use	The percentage of correct cross-validated predictions was 55% to 76% But, *p* < .02 in favor of the benchmark models
	[29]	Gloor et al. (2018)	Happiness and activation are negatively correlated with heart beats and with the levels of light. People tend to be happier when they are moving intensely	Average BPM heart: r = − .174, *p* < .01* Light level: r = − .111, *p* < .01* Activation: r = .460, *p* < .01*
	[35]	Zhang et al. (2018)	MoodExplorer can infer user’s compound emotion with exact match of 76.0% on average	

-.: No statistical test. r: correlation coefficient, FE: fixed effect estimate, β, B or b: (standardized) regression coefficient, adjd R^2^: adjusted explained variance, MD: mean difference, OR: odds ratio

Using the checklists for EMA studies from Liao et al. (2016) and van Roekel et al. (2019), we checked the risk of bias across the studies. Regarding the procedure and methods, 49 of the 53 studies reported on the type smartphone used, all studies reported on the prompt design, study duration, number of assessments per day and incentives provided and all but one study reported on the total number of items per assessment. Of the 53 studies, 37 reported the attrition rate, i.e. how many participants dropped out of the study. Fourteen of the 53 studies reported the average response time (time lag between the prompt and response) of the participants and as already noted, the overall compliance is only reported in 25 of the 53 studies and reasons for noncompliance are often lacking. In a few studies, participants were excluded based on compliance rates. Only three reviewed studies (25, 27, 31) reported a power analysis prior to the data collection to determine their needed sample size and reach a power of 0.8. One study performed a post-hoc power analysis (36) and concluded that their power was really low (0.18 instead of 0.8). A lot of studies miss data on compliance and all details of the design. Therefore, the reporting of the included EMA studies is often not complete and this indicates some risk of bias and selective reporting.

An often-reported limitation in the reviewed studies is the limited sample size in number or generalizability to the whole population. Samples in studies that use free available applications seem to be biased by self-selection. For example, 35 studies reported a bias in their sample, having attracted a younger (k = 10), more highly educated (k = 10), a mostly female (k = 21) or male (k = 6) sample compared to the whole population, with 12 studies having multiple biases, affecting the generalizability of the results. In addition, part of the applications (k = 25) were only available for iPhone or Android smartphones. This biased sample might affect the results and outcomes of the studies.

Another frequently reported limitation is controlling for only a small number of environmental variables (e.g. green/urban, noise or light levels or social environment) and other confounders (e.g. personal characteristics or weather). Since EMA measures behavior and experiences in real life, there is less control over the measurement and context. Confounders such as the weather, social environment, nutritional status, alcohol consumption, but also personality traits, might have affected well-being instead of the variable of interest.

Some studies (2, 5, 6, 36) reported their sampling strategy as limitation, since the number of data points and compliance levels were lower than expected. A way to solve this is to trigger questionnaires not only based on time, but based on variables of interest, such as location or activity. Lastly, another often reported limitation is the impossibility to determine the causality between the variable of interest and happiness with the current design and available data.

### Findings of Studies Using Other EMA Data Collection Devices

The search to EMA studies that used palm tops, PDAs or other EMA devices to assess well-being multiple times a day resulted in 8 studies that included healthy participants (Dvorak et al. 2018; Elavsky et al. 2016; Ilies et al. 2010; Johnston et al. 2013; Juth et al. 2015; Shiffman et al. 2002; South and Miller 2014; Yip 2005) (see supplementary data Sheet 3 for details of the studies and the results).

To summarize, seven of the eight studies were conducted in the USA, with the remaining study in the United Kingdom. The average number of participants included was 142.9 (SD = 85.8) with a range from 62 to 304 participants. The participants were on average 31.7 years old (SD = 13.9) and more females than males were included (68.1%, SD = 20.1). All studies used a measure of positive affect (e.g. PANAS) in their assessments. Positive affect was assessed in different contexts, namely in relation to smoking, stress, ethnicity, emotional coping, sedentary behavior and blood pressure. Three studies used a personal digital assistant (PDA), four used a palm-top computer and the last study used a hand held computer to deliver the prompts and assessments. The average study duration was 8.5 days (SD = 4.3) with an average of 5.4 prompts per day (SD = 1.4). All studies used time-contingent designs, but in the two smoking studies participant were also asked to answer questions after smoking a cigarette, i.e. event-contingent. Only two of the eight studies added objective data, either using an accelerometer to measure sedentary behavior or a cardiovascular monitor to assess blood pressure. Across the 8 studies, the average compliance rate was 78.4% (SD = 8.3) with a range from 70 to 91%.

Shiffman et al. (2002) found no relation between momentary positive affect and smoking, whereas Dvorak et al. (2018) reported an association between momentary positive affect and momentary smoking. Single studies found a negative association between momentary positive affect and stress and internalizing problems (South and Miller 2014) and sedentary behavior (Elavsky et al. 2016). Other studies found a positive association of positive affect with ethnic salience (Yip 2005), emotional approach coping (Juth et al. 2015), and work effort and demands in nurses (Johnston et al. 2013). Finally, there was no correlation between momentary positive affect and blood pressure (Ilies et al. 2010).

## Discussion

We performed a systematic review on smartphone-based ecological momentary assessments in well-being research in healthy participants. Using the PRISMA guidelines we retained 53 studies (out of 398 studies), which we included in the present review. Overall, the study designs were very heterogeneous of nature, with varying sample size, used questionnaires, phones, study duration (*M* = 12.8 days with most studies lasting seven days) and how often well-being was assessed during the day (range of 2–12 times a day). Additionally, well-being was assessed in relation to different (environmental) variables, from the (natural) environment, to physical activity, work and other variables. In addition to self-report data, half of the studies included some objective data measured using the smartphone sensors or additional meters (e.g. GPS, accelerometer data or telephone use). Less than half of the studies (47.2%) reported the response rate and compliance. Based on this limited information, on average 71.6% of the EMAs is responded to by the participants.

Based on the reviewed studies we can conclude that momentary well-being fluctuated daily and weekly, with higher well-being in the evening and weekend. These fluctuations disappeared when location and activity were included. On average, being in a natural environment and physical activity relates to higher well-being. Working relates to lower well-being, but workplace and company influence well-being. Besides the mentioned limitations before (e.g. relatively homogenous samples and less environmental control), a main limitation of the reviewed studies is the focus on average scores across people, and ignoring individual differences and patterns over time.

There are some notable differences between the designs of EMA studies with other data collection devices and smartphone-based EMA studies to well-being. First, the maximum sample size in smartphone-based EMA is much larger than in palm top or PDA studies. Next, the average study duration is longer when using smartphones (12.8 days) compared to other devices (8.5 days). These differences show that data collection is easier when participants can use their own devices. There are no (financial) restrictions on the both the sample size and study duration as researchers do not need to buy devices. Furthermore, smartphone studies more often add objective data to their designs, reflecting the flexibility of smartphone applications compared to other devices. Based on the limited number of studies, the compliance rates of both sets of studies are similar (*p* = 0.204), namely 71.6% for smartphone research and 78.4% for other devices. Only sedentary behavior is assessed in relation to well-being in both smartphone studies and a PDA study. In most studies, momentary positive affect is associated with less sedentary behavior. The other results cannot be compared.

Our review shows that smartphone-based EMA designs are feasible, have multiple advantages over other EMA collection devices and can be used to inform our understanding of well-being in addition to traditional questionnaire research. The real-time responses in a natural environment for the participants give an insight in the fluctuations and patterns of well-being in individuals that cannot be captured with retrospective self-report measures. Furthermore, assessing well-being in different contexts and/or in response to activities or events, can contribute to understand the dynamic nature of well-being and to identify the causal influences on well-being. This might be useful in creating interventions to increase well-being if we know what makes people happy.

### Guidelines for the Use of Smartphone-Based EMA Designs in Well-Being Research

Based on findings of the reviewed studies and the current limitations we propose recommendations and future directions for EMA studies to get a better hold of the complexity of well-being (see Table 5 for an overview).

**Table 5:** Guidelines for future smartphone-based EMA research of well-being

Design issue	Guidelines
Sample	Depends on the goal A large anonymous sample: widely advertise a free downloadable app Target sample: invite participants with certain characteristic and send a link to download the app
WB measure	Include questions on both the affective and cognitive part of subjective well-being Use a few WB questions, instead of a full scale to prevent answering becoming too repetitive
Objective data	Include passive and objective sensor data in addition to self-report when possible Use the new developments in technology to link self-report data to objective environmental data
Application	If possible, develop the app for both the Android and iOS platform Test the app multiple times Offer feedback on the participants well-being levels to make participation fun, after data collection is complete, to avoid reactivity problems
Study duration	Depends on the goal and context of interest, but may range from a few days – up to a month Include all days of the week to assess weekly fluctuations of WB Include EMA in different seasons to assess seasonal WB fluctuations
Number of prompts per day	Depends on the time-scale of variation of WB in the context of interest On average, prompting 2 times per day up to every hour can be reasonable If possible, base the number of prompts on individual differences or let participants decide their number of prompts
Compliance	Be active to keep compliance levels high Use subject-management procedures as incentives or training Limit the latency between prompt and answer
Analyses	Focus on fluctuations and patterns of well-being and other experiences/behaviour instead of the average or sum Investigate the individual differences of well-being and the relation with environmental variables
Report	Report compliance levels and relate this to study characteristics Follow the report guidelines of Liao et al. (2016) for EMA studies Use the checklist of van Roekel et al. (2019)

As a first guideline, we recommend to use smartphones instead of other data collection devices in future EMA studies. Whereas you need to provide participants with the other devices, nearly everyone nowadays owns a smartphone (already more than 70% of the Western and USA population and 45% worldwide (GSMA intelligence, 2019)). This leads to the possibility of reaching larger samples and longer study durations are possible. In addition, the flexibility and continuing developments of smartphones and applications is preferable when designing strong studies, i.e. the addition of objective data collection is easier. Only in studies where the target sample is expected not to have a smartphone, e.g. younger children, providing a device (either a smartphone or any other device), could be a good solution.

#### Sampling

An important advantage of smartphone-based EMA designs is the possibility of reaching many people and including unique or large samples. Advertising the nation- and worldwide applications *Track Your Happiness* and *Mappiness* resulted in the largest sample sizes ever used in EMA research (N > 10.000). This review shows that a smartphone-based EMA design is feasible to use in all types of samples, ranging from adolescents to older participants. Vilaysack et al. (2016) showed that it is even feasible to perform smartphone-based EMA research in 5–7 year old children. With the increasing rate of smartphone users around the world and simple design of an application, more people can be reached in a smartphone study that are otherwise not included in research. Using such large and heterogeneous samples, the influence of and interplay with many factors (e.g. in culture, education, environment) can be investigated on a large scale.

Whereas some reviewed studies advertised such a freely available application, most studies recruited a convenience or specific target sample (e.g. employees or university students). Both recruitment ways have advantages and the best recruitment depends on the study goal. An anonymous sample can become really large and leads to more power, but there is more control over a targeted sample (e.g. specific characteristics or to contact them for follow-up). Large scale open recruitment can have the same drawback as large online survey studies, such as oversampling of females (Saleh and Bista 2017). A quantity-quality tradeoff can be seen in recruiting an anonymous larger sample versus a smaller sample with more control over the representativeness (similar to the quantity-quality trade off in a genome-wide association study (Okbay et al. 2016). We recommend to choose the recruitment method based on the specific research question and goal.

Only three reviewed studies reported a power analysis to estimate the required sample size for their study prior to data collection. A power analysis to calculate the required sample size in EMA studies is often complicated, since power in EMA studies does not only depend on the number of participants, but also on the number of prompts per day. The choice to increase power based on the number of participants or prompts depends on which level the effects are of most interest. Mathieu et al. (2012) suggest that when interested in detecting lower level effects (e.g. relationship between environment and well-being) maximizing the number of lower-level units (number of prompts) is most beneficial, whereas when interested in higher level effects, (e.g. etiology of the within person fluctuations of well-being) increasing the sample size might be more beneficial.

#### Measures of Well-Being

Most reviewed studies used one happiness question or a combination of positive affect adjectives to assess subjective well-being (e.g. relaxed, feeling good). Only one study used a full well-being sale, the Warwick-Edinburgh Mental Well-being Scale with 14 items (Bakolis et al. 2018). Answering many questions multiple times a day might become too much of a burden for participants and could decrease the compliance and data quality. For example, in the study of Bakolis, only 25 of the 108 participants had a compliance rate of 66% or higher. Using a full questionnaire to investigate momentary well-being is thus not recommended. Krueger and Schkade (2008) investigated the reliability of a single *momentary* affect measure (e.g. happy, depressed, angry) in the Day Reconstruction Method (DRM) and found reliabilities of 0.50–0.70 (similar to the reliability of a *general* well-being measure (Diener et al. 1985; Lyubomirsky and Lepper 1999). As DRM and EMA designs result in nearly identical happiness ratings over the day, single item measures are also thought to be reliable in EMA research (Dockray et al. 2010; Kahneman et al. 2004). Therefore, including only one or a only a few well-being questions is preferable to keep the participant burden low.

Whereas the reviewed studies only investigated the hedonic/affective part of well-being (e.g. happiness or mood), well-being also consist of a cognitive part (e.g. satisfaction with life) and eudemonic well-being (the fulfilment of human potential (Ryff 1989; Ryan and Deci 2001)). These components of well-being have not been assessed in smartphone-based EMA studies yet. Rating your current mood or happiness (i.e. affective WB) is relatively easy, whereas a bit more thinking and cognitive processing is needed to report on momentary life satisfaction, i.e. cognitive well-being. Nevertheless, reporting life satisfaction in the moment is possible and some variation over time is expected. In contrast, Steptoe (2019) suggest that answering questions on eudemonic well-being (the meaning of life), needs more thinking and cognitive processing, including aggregation over time and comparisons with self-selected standards. Less variance in daily or hourly eudemonic well-being is expected and therefore, this aspect of the well-being spectrum seems less suitable for EMA. We recommend to include questions in an EMA study to investigate fluctuations in both cognitive and affective well-being (e.g. “*How do you feel right now*?” (unhappy/happy) and “*How satisfied are you with your life at this moment*?” (unsatisfied/satisfied)).

#### Objective Data

More than half of the reviewed studies combined self-report with objectively measured data, either collected from smartphone sensors or an additional (accelero-)meter. Including the measures on the same device (e.g. two different apps) or in the same application is most convenient for both the researchers and participants, as data is integrated immediately and participants do not have carry additional meters.

An important advantage of objective data in general is the passive nature of the data and thereby the ecological validity (i.e. data is collected without active participation of the participant in their natural setting). This reduces the participant burden, and complements self-report data, with no issues of compliance. Furthermore, objective data can provide more reliable information. For example, in physical activity research, only a weak correlation between self-report and objectively assessed accelerometer data has been found, indicating biases in self-report (Dyrstad et al. 2014; Prince et al. 2008). Also in sleep and phone/Internet use research, objectively assessed data might be more reliable than self-report (Boase and Ling 2013; Girschik et al. 2012; Junco 2013).

Combining self-report data and sensor data is a powerful design and can lead to new insights into the interaction of mental, physical, and environmental processes in daily life. Over the next years, objective data is likely to be included more often with the continuing developments in smartphone technology, e.g. more sensors are being embedded in mobile phones (step count or heart rate) (Mohr et al. 2017). Recently, open source platforms have been developed to make the collection of passive data from smartphone sensors easier for researchers, facilitating the use of passive data in research even further. A few examples are the AWARE framework (Ferreira et al. 2015), RADAR-base (Ranjan et al. 2019) and the Insight app (Montag et al. 2019).

However, some difficulties with passive data collection remain, as also noted by Mohr and colleagues (2017). First, smartphone sensors vary in different phones and personal characteristics, such as age and gender, might affect the data. Older people use their phones differently than younger people and males more often carry along their phones in their pockets compared to females (Ichikawa et al. 2005). To correctly process and interpret objective data from different phones and populations more research is needed. Furthermore, with the new European General Data Protection Regulation (https://eugdpr.org) in action, researchers have to adhere to strict rules regarding privacy and personal data. Passive data results in large amounts of possibly identifying information, data processing is needed before storage. For example, GPS data cannot be stored with all details to prevent identifying information on the home location, but should be measured using an unknown relative location or transformed to an appropriate level (e.g. street level).

#### Schedule and Prompting Strategy

The average number of prompts per day in the reviewed studies on well-being is 5.0 (range: 2–12) and the duration 12.8 days. According to an earlier review of EMA, it is reasonable to administer up to 10 EMAs a day to participants for periods ranging from 1 week to 1 month (aan het Rot et al. 2012). Our review shows that the frequency of prompting and the study duration is influenced by the specific study goal and context. To capture fluctuations, the number of prompts needed depends on the time-scale of variation in the context of interest. When interested in more varying events or contexts, participants need to be prompted more often to capture all types of the context. For example, Poerio et al. (2013) prompted participants 12 times per day to investigate the effect of different types of mind wandering on well-being, whereas three or four prompts per day seem to be enough to assess well-being in different environmental contexts (e.g. Bejarano et al. 2019; MacKerron and Mourato 2013). With rare events, event-based sampling or a combination of different sampling strategies might be preferred to capture these events and reduce the number of prompts.

Based on these findings and the literature on well-being, we recommend to prompt participants based on the context of interest, but at least three times a day (every morning, afternoon and evening) for a minimum of seven days to assess the fluctuations of momentary well-being over the day and on different weekdays. In addition, based on findings of seasonal depression (American Psychiatric Association 2013) and individual differences in mood changes in summer and winter (Golder and Macy 2011; Klimstra et al. 2011), we might expect seasonal fluctuations in well-being or a different pattern of daily WB fluctuations. Therefore, we recommend to assess well-being in the different seasons using EMA as well.

A few studies let the participant decide how many prompts he/she wanted to receive, with a minimum or default level. This individual control over the number of prompts might increase the compliance levels. Unfortunately, with the limited reported data, we could not test this relation. In addition, individual differences might exist in the preference for and reaction to the number of prompts. More research is needed to assess this preference in combination with participant burden and compliance levels. Until these limitations are solved, we recommend to base the number of prompts on the time-scale of variation in well-being in the context of interest.

#### Applications

Most studies used applications specifically developed for the research question and context of interest. As the development of a properly working application cost a lot of time and money, collaborations with app developers or other researchers should be encouraged to create an app combining research, good usability and technical factors (McEwan et al. 2019). The application should be tested multiple times to reduce the burden for participants, since participants drop out as soon as a problem occurs or an update is needed (McEwan et al. 2019).

Furthermore, especially when not offering incentives, providing feedback to participants after the study (e.g. in the form of a well-being graph over time) is recommended to make participation fun and keep compliance levels high. For example, in the Mappiness studies (MacKerron and Mourato 2013), participants received personalized graphs with happiness over time, related to where they were, with whom and what they were doing. However, undesirable for research and EMA studies, this feedback may make participants aware of their well-being, leading to a reactive effect and influencing the data. Therefore, feedback should be provided after data collection is completed. Awareness of well-being might lead to people initiating more activities that make them happy. Ludwigs et al. (2018) did show that two weeks of paying more attention to your well-being has a small positive effect well-being. However, recently De Vuyst et al. (2019) showed that 10 EMAs of emotions per day does not impact participants’ emotional experience over time. Both samples consisted only of (psychology) university students. More research in a more heterogeneous sample to this reactive effect of attention to well-being is necessary for a conclusive answer.

#### Response and Compliance

Not even half of the studies reported the response rate and compliance of the participants. Based on the limited data, the EMA compliance was relatively high, with a mean of 71.6%, but most studies did not reach the preferred 80% compliance to have generalizable data of daily life (Stone and Shiffman 2002). Furthermore, the kind of missing data (random vs. not random) is not reported in the reviewed studies, but should be taken into consideration. Based on the limited reported data, results did suggest there is no difference in compliance using a research smartphone or using the participant’s own smartphone. In addition, we did not find a clear relation between compliance and other study characteristics, such as the study duration, number/timing of prompts, providing incentives or participant characteristics, similar to Jones et al. (2019). Further research is needed to confirm these findings, since this information is critical to optimize future EMA studies.

Only three studies reported the latency (time difference) between the prompt and answer. This latency is important, since the validity of EMA studies is based on momentary experiences, i.e. answering of questions should be in the moment. Though most studies did limit the answering time, this could be up to an hour, reducing the ecological validity. However, returning to the added value of passive data, if you know the timing of the answer and passive data collection is continuously, the time difference between prompt and answer might be less of a problem.

To keep compliance levels high, subject-management procedures such as incentives, but also training and feedback might help. For example, incentives for completing assessments are found to increase compliance levels to some extent in earlier EMA studies (e.g. in substance abusers Beckham et al. 2008; Shiffman 2009; Sokolovsky et al. 2014)). However, this evidence is limited to non-smartphone EMA research. In our reviewed smartphone studies, compliance levels seemed to be independent of incentives. Further research to compliance and incentives in smartphone-based EMA is needed.

#### Analyses

Whereas most studies did apply a multilevel model to the EMA data, a main limitation of the reviewed studies is ignoring the variance (individual differences) and within person patterns over time. Focusing on group averages is common in the field of wellbeing (and beyond), but this leads to missing important information about individual differences. In EMA research, ignoring individual differences also reduces the ecological validity (Ram et al. 2017). As individuals differ in their patterns of mood, behavior, and other experiences in life, the average person or pattern of those variables across participants does not exist in the real world. For example, in contrast to the general belief that people feel better after physical activity, strong individual differences in the affective responses during and after exercise are found (Ekkekakis et al. 2005; Rose and Parfitt 2007, Welch et al. 2007). In the study of Van Landuyt et al. (2000) half of the participants felt less happy during and after physical exercise compared to before. Schutte and colleagues (2017) showed that genetic factors explain 15% of the individual differences in affective responses (Schutte et al. 2017).

The same individual differences might exist for well-being and other environmental variables, such as location preferences or outdoor activities. For example, MacKerron and Mourato (2013) show that people, *on average*, are happier in natural environments. However, individuals do differ in their reaction to urban or natural environments. Newman and Brucks (2016) showed that people differ in the environment needed to restore their self-control. People high on neuroticism prefer an urban environment to restore self-control, whereas people low on neuroticism prefer a more natural environment. This suggests that individual differences in the effect of natural environments on well-being might exist as well.

Another limitation of averaging data is ignoring a large part of the data. Patterns of moods or behavior over the day or week contain a lot of information and might be more informative than a total or average score. As mentioned in the introduction, when investigating the relation between health and physical activity, the pattern of accumulation and not the total volume is more informative (Chinapaw et al. 2019). As another example, Smit et al. (2019) tried to predict an increase in depressive symptoms in six patients. Results show a rise in restlessness more than 2 months before the increase in depressive symptoms, whereas the total negative affect and positive affect scores did not have the same capacity to signal future increase in depressive symptoms.

In summary, we recommend to focus on fluctuations, patterns of well-being, and individual differences instead of the average or sum. In addition, EMA data is perfect to investigate individual differences in well-being and the relation with environmental variables or other (causal) correlates.

### The Future of EMA in Well-Being

Well-being has become a popular research topic with increasing number of publications in the last years. Well-being findings have the potential to inform policy and are applied more often in the society. Nowadays, using new technologies, detailed information about the fluctuations in well-being can lead to more insight in what people experience in their daily life and what makes them happy, from day to day or even hour to hour.

Our review shows that using a smartphone application to measure well-being multiple times a day is feasible and preferable over other EMA data collection devices. More systematic research on the fluctuations of the different components of well-being (e.g. affective and cognitive WB) in diverse contexts is needed to understand what influences momentary well-being and makes people happy. The continuing developments in smartphone technology (e.g. processing power, battery life, and new sensors and functions) and open source platforms will result in easier collection of self-report and objective data (Harari et al. 2016). The addition of objective data to self-report data is especially valuable to understand the relation between context, environmental variables, and momentary well-being. We recommend to use all aspects of EMA data and to focus on data patterns and individual differences using sophisticated analyses such as time-series analyses.

In the future, when the fluctuations and patterns of well-being and the interaction with environmental variables that make individuals happy are known, a next step in smartphone-based well-being research is a shift from EMA (assessing behavior in the moment) to EMI (ecological momentary intervention), intervening on behavior in the moment to increase well-being (Heron and Smyth 2010). For example, the Shmapped application (McEwan et al. 2019) is developed as both a data collection and intervention tool. When prompted, participants are instructed to notice the nature around them and to recall good things. Preliminary results show improvements in wellbeing at one-month follow-up (McEwan et al., *under review*). Furthermore, a recent meta-analysis showed that smartphone-based interventions (e.g. awareness or relaxation exercises) to increase well-being in clinical samples have a small positive effect on the quality of life (Versluis et al. 2016). However, most interventions use a one size fits all approach. All participants receive the same intervention to increase well-being and individual differences and reactions to the intervention are not responded to. Taking into account individual differences might increase the effectiveness of future well-being interventions.

## Limitations

A quantitative meta-analysis on the fluctuations of well-being or the effect of environmental variables was not possible, since the studies were too heterogeneous in study methods, contexts and analyses (e.g. the well-being measure, duration, number of prompts, reported statistics). Another limitation of this review might be the incomplete retrieval of articles. The field of smartphone and application based EMA research is relatively new and gaining popularity. The terminology to describe EMA studies varies and the results of EMA studies are being published in various forms and places. To be as conclusive as possible, we followed PRISMA criteria for systematic reviews.

## Conclusions

The 53 reviewed studies showed that a smartphone-based form of EMA research is feasible to assess the fluctuations of momentary well-being in different contexts. Based on the used designs and findings, we provided recommendations for future smartphone-based EMA research in well-being. In the near future, with the continuing developments in smartphone technology, the use of smartphone applications combining EMA self-report and objective data can result in more specific knowledge about the fluctuations of well-being and what makes people happy.

## Electronic supplementary material

Below is the link to the electronic supplementary material.

Supplementary file1 (XLSX 116 kb)
